# Effects of Adding Punicalagin or Oleuropein to TRIS Diluent on Quality of Frozen–Thawed Semen from Rams

**DOI:** 10.3390/ani15091242

**Published:** 2025-04-28

**Authors:** Mohamed Shehab-El-Deen, Mohamed Ali, Ibrahim Alolayan, Abdullah Aljuaythin, Yasser Alrauji, Soliman Aldobaib, Shaaban S. Elnesr

**Affiliations:** 1 Department of Animal and Poultry Production, College of Agriculture and Food, Qassim University, Buraydah 51452, Al-Qassim, Saudi Arabia; m.shehabeldeen@qu.edu.sa (M.S.-E.-D.); alym@qu.edu.sa (M.A.); ibrahimalialolayan@gmail.com (I.A.); 451116528@qu.edu.sa (A.A.); raojy@qu.edu.sa (Y.A.); s.aldobaib@qu.edu.sa (S.A.); 2 Department of Animal Production, Faculty of Agriculture, Suez Canal University, Ismailia 41522, Egypt; 3 Department of Poultry Production, Faculty of Agriculture, Fayoum University, Fayoum 63514, Egypt

**Keywords:** sheep, semen cryopreservation, extender, antioxidants, punicalagin, oleuropein

## Abstract

This study aimed to investigate the effects of adding different levels of punicalagin or oleuropein to TRIS diluent on the quality of frozen–thawed semen from Najdi rams. Semen was diluted using TRIS-based diluter with 15% egg yolk (control group); supplemented with 0.1, 0.5, or 1 mg/100 mL punicalagin (in Experiment 1); or supplemented with 1, 2.5, or 5 mg/100 mL oleuropein (in Experiment 2). The collected semen was evaluated and cryopreserved, with the motility and concentration of sperm assessed using a CASA system. The results showed that the total motile spermatozoa (TMS), percentage of progressive motile spermatozoa (PMS), curvilinear velocity (VCL), rectilinear velocity, average path velocity (VAP), linearity coefficient, straightness index, minor defects, and sperm vitality were higher in the 0.1 mg/100 mL punicalagin group (*p* < 0.05) than in other groups. HOST% post-thawing was significantly higher in all punicalagin groups compared to the control group (*p* < 0.001). The percentages of PMS, TMS, VCL, minor defects, and sperm vitality were higher in the 1 mg/100 mL oleuropein group (*p* < 0.05) than in other groups. Oleuropein supplementation at 5 mg/100 mL decreased VAP in cooled sperms, while all levels increased VAP post-thawing. HOST-positive sperms% post-thawing was higher in all oleuropein-treated groups than the control group (*p* < 0.001). Moreover, oleuropein nonsignificantly increased the acrosome integrity in cooled sperms, while higher studied concentrations of oleuropein (2.5 and 5 mg/100 mL) decreased the acrosome integrity in frozen sperms. In conclusion, adding punicalagin (0.1 mg/100 mL) or oleuropein (1 mg/100 mL) to TRIS diluent improved the quality of frozen–thawed semen from rams.

## Simple Summary

The sensitivity of ram sperm to reactive oxygen species is high. The supplementation of antioxidant-rich diluents may help to protect sperm against oxidative damage. This study aimed to evaluate the role of adding natural antioxidants (punicalagin or oleuropein) as a supplement to the basic TRIS extender on the post-thaw quality parameters of spermatozoa from Najdi rams. The results exhibited that the addition of punicalagin or oleuropein to the diluent could boost the quality of ram sperm through improving the viability, total motility and progressive motility, and patterns of motility in the considered spermatozoa.

## 1. Introduction

Artificial insemination is one of the most used assisted reproductive techniques in livestock—especially sheep—and has facilitated the effective use of genetically superior rams. Effectively freezing ram semen is a key factor in the progress of the artificial insemination field. Through the freezing of semen, superior genetic resources can be well preserved [1,2]. Semen cryopreservation and artificial insemination have potentially contributed to improved selective breeding with favorable traits [3,4]. However, the effective benefits of semen cryopreservation have some restrictions, particularly due to the exposure of spermatozoa to freezing–thawing and oxidative stresses, which harmfully affect the plasma membrane structure and function of spermatozoa and, ultimately, their survival after freezing [5,6]. During the freezing of semen, the spermatozoa are exposed to cryoinjury. To protect sperm cells from cold shock, cryoprotectants are routinely added to increase their resistance to thermal changes [7,8]. Specifically, ram sperm has been identified as being more sensitive to cryo-damage than that of other animals [5], which may be related to the high levels of polyunsaturated fatty acids (PUFAs) in the plasma membrane of ram spermatozoa [9]. Furthermore, high PUFA levels cause sperm cells to be more sensitive to the lipid peroxidation process in the presence of reactive oxygen species (ROS) [10].

During cryopreservation, sperm cells are faced with certain stressful conditions other than freezing, such as oxidative and osmotic stresses, which may decrease the quality and viability of sperm cells after freezing [11,12]. Oxidative stress has been reported to hinder the cryo-resistance of sperm due to plasma and acrosome membrane impairment and nuclear damage [13]. The mitochondria regulate oxidative processes through the amount of ROS produced in the sperm cell. To attain successful fertilization, the sperm cell should have a sufficient concentration of antioxidants [14]. However, when the antioxidant concentration does not match the formation of ROS, oxidative stress occurs. Therefore, oxidative stress suppresses the quality and viability of sperm cells [15]. It has been extensively stated that such high levels of ROS formation could be equalized by adding certain antioxidants to the semen extenders. Natural antioxidant additives are considered more favorable in this regard [16]. In particular, oleuropein and punicalagin are natural antioxidant substances that can scavenge free radicals.

Oleuropein is a polyphenolic component which is enriched in the oil and leaves of olive plants, possessing the ability to both scavenge nitric oxide and to increase the expression of inducible nitric oxide synthase in cells [17]. Oleuropein has effective antioxidant [18] and antimicrobial [19] activities. Other studies concluded that supplementation of extender with oleuropein (olive fruit extract; rich in oleuropein) enhanced post-thaw semen quality in buffalo [20] and human (Olea europaea leaf extract) [21] and cold storage semen quality in boer [22]. However, further research on the role of the protective action of oleuropein on ram semen is required in order to determine the best concentrations and to develop oleuropein as a potent diluent supplement in the cryopreservation of Najdi ram semen.

Punicalagin is the major antioxidant polyphenol ingredient in pomegranate (*Punica granatum* L.) fruits, which has been shown to have protective effects on the semen parameters of buffaloes when added to tris-based extenders [23]. It has been demonstrated that adding pomegranate peel methanolic extract (0.48 mg/mL) to Tris-based ram semen extender protects the properties of sperm post-thaw by enhancing biochemical profiles and antioxidant capability [24]. Similarly, pomegranate juice significantly increased sperm motility, membrane integrity, viability, function, and velocity; decreased total sperm abnormalities; and enhanced antioxidant capacity, as indicated by the integrity and viability of cryopreserved Ossimi ram spermatozoa [25]. Accordingly, adding punicalagin to ram extender may increase the viability and quality of frozen sperm, considering its antioxidant properties.

From the above, using antioxidants in ram semen diluent appears to be a promising solution to improve the quality of semen from various animals. Therefore, after reviewing the existing literature, the current study aimed to evaluate the effects of adding natural antioxidants (punicalagin or oleuropein) as a supplement to the basic TRIS extender on the post-thaw quality parameters of spermatozoa from Najdi rams.

## 2. Materials and Methods

All experiments were carried out at a private Najdi sheep farm located in Buraidah, Qassim region, and the Artificial Insemination Laboratory of the Department of Animal and Poultry Production, College of Agriculture and Food, Qassim University, Qassim region, Kingdom of Saudi Arabia.

### 2.1. Experiments and Animals

Six fertile Najdi rams, which were free of health problems and aged between two to four years, were subjected to semen collection. All animals were housed in an open shelter, and clean water, concentrates, and alfalfa hay were offered ad libitum. Mineral cube salts were also offered to the animals. All semen samples (12 replications) were pooled together and allocated to studied extenders including control group.

### 2.2. Semen Collection

**Figure 1:**
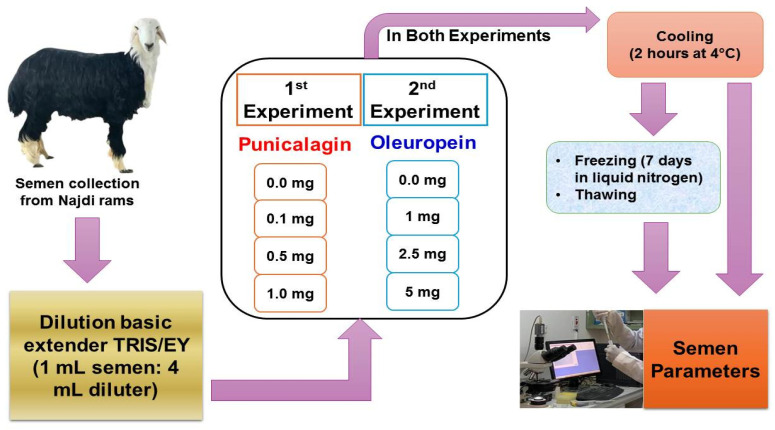
Experimental design of the current study.

An ordinary small ruminant artificial vagina was used for semen collection. Semen samples were obtained from the rams 2 times per week for 6 consecutive weeks (12 replications). The collected semen was kept at 37 °C in a water bath. All samples were analyzed on site and only samples with at least 70% total motility were used. A computer-assisted semen analysis (CASA) system was used for assessing semen quality in the laboratory within 1 h of collection. Semen was assessed for quality measures including color, pH, ejaculate volume, percentage motility, total motility, viability, and concentration. Sperm quality parameters were evaluated using a computer-assisted semen analysis system (CASA: ISAS^®^ program, Proiser R + D, Valencia, Spain). The experimental design of the current study is shown in Figure 1.

### 2.3. Experiment 1: Punicalagin Supplementation

Punicalagin was purchased from Sigma-Aldrich^®^, Saint Louis, MO, USA. Semen was diluted using TRIS-based extender with 15% egg yolk (EY) (control group). TRIS extender preparation was carried out as follows: buffering agents (3.643 g TRIS and 1.99 g citric acid), 0.5 g glucose, 15 mL EY and 5 mL glycerol. Non-pyrogenic water was added up to 100 mL. The TRIS extender was supplemented with 0.1, 0.5, or 1 mg/100 mL punicalagin.

### 2.4. Experiment 2: Oleuropein Supplementation

Oleuropein was purchased from TCI Chemicals for Laboratories and Production, Philadelphia, PA, USA. Semen was diluted using TRIS-based extender with 15% EY (control group) or supplemented with 1, 2.5, or 5 mg/100 mL oleuropein.

### 2.5. Cryopreservation

All ram semen samples were reconstituted with the TRIS diluter (1 mL semen + 4 mL TRIS diluter). After cooling (2 h at 4 °C), extended semen was assessed using the CASA system and then filled in 0.5 mL straws. The concentration of frozen spermatozoa was 60 million per straw. The cryopreservation procedure was as follows: loaded straws were placed horizontally above the liquid nitrogen surface by 3 to 4 cm for 20 min for sensitization via the vapor of liquid nitrogen, then submerged in liquid nitrogen.

After seven days of freezing, the straws were thawed directly in a water bath (38 °C/60 s) and then examined using the CASA system [26].

### 2.6. Assessment of Sperm Motility

All semen was assessed post-cryopreservation for the pattern of motility using the ISAS^®^ v1 software (Valencia, Spain). Spermatozoa motility parameters were assessed in seven successive images from different fields with a magnification of 10× and negative-phase contrast. A minimum of 300 spermatozoa/sample were examined. Spermatozoa motility was immediately documented, in terms of total motile sperm (% TMS), progressive motility spermatozoa (%), curvilinear velocity (VCL; μm/s), rectilinear velocity (VSL; μm/s), the average path velocity (VAP; μm/s), linearity coefficient (% LIN) (LIN = [VSL/VCL] × 100), and straightness index (% STR) (STR = [VSL/VAP] × 100). Wobble of the curvilinear trajectory (WOB) (WOB= [VAP/VCL] × 100). The frozen straws underwent a thawing process by being immersed in a water bath at a temperature of 38 °C for 60 s. Subsequently, the contents of the straws were evacuated into a tiny tube that had been warmed. The ISAS program was used to test the total and progressive motility of the samples. Spermatozoa with VAP values < 10 μm/s were considered immotile. However, spermatozoa with VAP > 20 μm/s, VSL > 30 μm/s, and VCL > 45 μm/s were considered motile [27].

Parameters such as LIN, STR, and WOB help assess the effectiveness of sperm motility, which impacts semen quality and fertilization success. Thus, analyzing these parameters can help predict the ability of sperm to reach and fertilize the egg. The WOB measures the extent to which sperm motility oscillates around its trajectory. However, indicates the degree of “wobbliness” or irregularity in sperm motility. A high WOB indicates irregular or inefficient motility, while a low WOB reflects straighter, less wobbly motility, enhancing motility efficiency and fertilization potential. Furthermore, WOB reflects the stability of motility [28,29].

### 2.7. Assessment of Spermatozoa Morphology, Plasma Membranes Functionality and Acrosome Integrity

**Figure 2:**
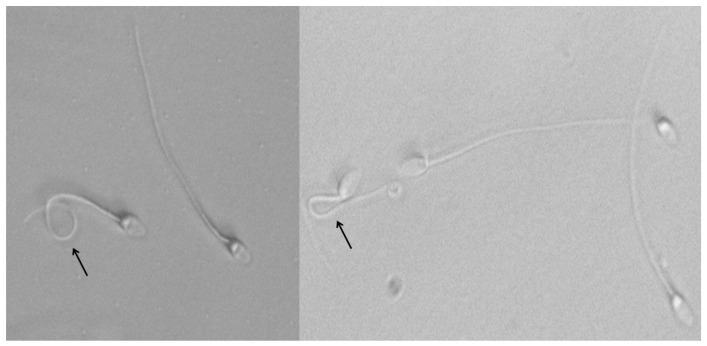
Plasma membrane functionality of spermatozoa evaluated by hypo-osmotic swelling test (HOST). Swollen sperm (coiled tail) indicates a functional and intact plasma membrane (black arrows).

Assessments of plasma membrane functionality acrosome integrity and spermatozoon morphology were performed as follows: The hypo-osmotic swelling test (HOST) was carried out to assess the functionality of the sperm plasma membrane. The method of Fonseca et al. [30] was followed with minor modifications; briefly, this method is based on a fructose-based solution with 125 mOsmol at 37 °C (2 mL), to which 20 μL of the different semen samples were added. After incubation for 50 min in a water bath at the above-mentioned temperature, spermatozoa were checked to determine whether the sperm tail was coiled or not (Figure 2). Approximately 100 spermatozoa were assessed under phase-contrast microscopy with 400× magnification.

For acrosome membrane integrity, the method of Hafez [31] was followed with minor modification. Acrosome integrity was evaluated using the Giemsa staining procedure [31]. One drop of diluted samples was mixed on a microscope slide with one drop of isosmotic 0.2% Trypan blue [prepared by mixing 0.4% trypan blue SIGMA T-8154 (Merck, Darmstadt, Germany) and 0.9% NaCl (1:1, *v*/*v*)] and were smeared. After vertical air-drying, the slides were fixed for two minutes with a fixative composed of 86 mL of HCl (1.0 N), 14 mL of 37% formaldehyde solution, and 0.2 g Neutral red and then were rinsed with tap and distilled water, respectively. The smears were stained in 7.5% Giemsa (SIGMA, GS-500, Saint Louis, MO, USA) for 3.5 h at 37 °C or overnight at room temperature. The slides were rinsed with tap water then distilled water and held for two minutes in a jar containing distilled water for better differentiation. Finally, the slides were dried in the air, and covered by coverslips. Two hundred spermatozoa were examined under a light microscope (×1000) to determine percentages of spermatozoa with defected acrosome. To assess the morphology of the sperm, we followed the procedure of Evans and Maxwell [32]; in this method, nigrosine eosin staining was used to determine normal spermatozoa. Abnormal spermatozoa with different defects were counted using light microscopy at 1000× magnification. The morphological defects of spermatozoa were categorized as sperm abnormalities based on their impact on fertility. Major defects encompassed a majority of abnormalities observed in the head and midpiece, as well as proximal cytoplasmic droplets and single abnormalities occurring at a high frequency. However, minor defects included looped tails, detached sperm heads, and distal cytoplasmic droplets [33].

Sperm vitality: fluorescent stains were used to assess sperm vitality. The fluorescent stains were acridine orange (AO) and propidium iodide (PI) and was provided by Halotech DNA, S.L., Madrid, Spain. Green fluorescence of head spermatozoa occurs when AO is retained within intact cells. PI stain can only bind to and stain cellular DNA in damaged cells, causing them to have red fluorescence. However, PI cannot penetrate living cells. A minimum score of 300 spermatozoa per sample was counted.

### 2.8. Statistical Analysis

The data were checked for normal distribution using the Kolmogorov–Smirnov test (SPSS, version 22). For comparison among treatments, one-way analysis of variance (ANOVA) was performed. The LSD test was used to compare among treatment groups. The differences were considered statistically significant if the *p*-value was less than 0.05.

## 3. Results

### 3.1. First Experiment Results

#### 3.1.1. Effects of Adding Punicalagin to Najdi Ram Semen Extender on Motility Kinetics and Characteristics in Cooled Sperms

The effects of adding punicalagin to the Najdi ram semen extender on the motility kinetics and characteristics of sperm in cooled sperms are presented in Table 1. The total motile spermatozoa and the percentage of progressive motile spermatozoa were higher in group P1 (0.1 mg/100 mL punicalagin) (*p* < 0.05 and *p* < 0.001, respectively), compared to the other groups. The VCL results indicated that the P1 group obtained the highest value regarding the punicalagin treatments (*p* < 0.001). Notably, the addition of punicalagin enhanced VSL, VAP, LIN, WOB, and STR at all studied concentrations (*p* < 0.001), when compared with the control.

#### 3.1.2. Effects of Adding Punicalagin to Najdi Ram Semen Extender on Sperm Morphology, Acrosome Integrity, and Plasma Membrane Functionality in Cooled Sperms

The results shown in Table 2 demonstrate no significant effects (*p* > 0.05) of adding punicalagin to the Najdi ram semen extender on the normal spermatozoa percentage, major defects, or acrosome integrity in cooled sperms; however, minor defects were slightly higher in the P1 group (0.1 mg/100 mL punicalagin. HOST-positive sperm% was significantly higher in the P3 group (1 mg/100 mL punicalagin), when compared to the other groups (*p* < 0.001).

#### 3.1.3. Effects of Adding Punicalagin to Najdi Ram Semen Extender on Motility Kinetics and Characteristics of Sperm Post-Thawing

The impacts of punicalagin supplementation to the extender on post-cryopreservation sperm motility kinetics and characteristics of sperm are presented in Table 3. The total motility was significantly increased at all studied concentrations of punicalagin, when compared to the control group (*p* < 0.001). The percentage of progressive motile spermatozoa was higher in the P1 group (0.1 mg/100 mL punicalagin) than in the other groups (*p* < 0.001). The VCL tended to be the lowest in the P2 group (0.5 mg/100 mL punicalagin; *p* = 0.091). The lowest punicalagin concentration used (0.5 mg/100 mL punicalagin) decreased WOB (*p* < 0.05). The findings indicated that supplementation of punicalagin at a concentration of 0.1 mg/100 mL increased VAP, LIN, (*p* < 0.01) VSL, and STR (*p* < 0.001).

#### 3.1.4. Effects of Adding Punicalagin to Najdi Ram Semen Extender on Sperm Vitality, Morphology, Acrosome Integrity and Plasma Membrane Functionality Post-Thawing

As shown in Table 4, concerning the morphology of sperm post-thawing, while the normal percentage of sperm, major defects, and acrosome integrity were not affected, the percentage of minor defects increased in all punicalagin groups (*p* < 0.05). Meanwhile, all studied concentrations of punicalagin showed a higher ratio in the HOST-positive sperm, when compared to the control group (*p* < 0.001). Sperm vitality was significantly improved with the addition of 0.1 mg/100 mL punicalagin (*p* < 0.001).

### 3.2. Second Experiment Results

#### 3.2.1. Effects of Adding Oleuropein to Najdi Ram Semen Extender on Motility Kinetics and Characteristics in Cooled Sperms

The effects of adding oleuropein to the Najdi ram semen extender on motility kinetic and characteristics in cooled sperms are presented in Table 5. The percentage of progressive motile spermatozoa tended to be higher in the O1 group (1 mg/100 mL oleuropein) than the other groups. VCL was significantly higher in the 1 mg/100 mL oleuropein group, compared to the control group (*p* < 0.05). However, the total motile spermatozoa, type of movement, and VSL were not affected by the addition of oleuropein. The VAP results indicated that the O3 group (5 mg/100 mL oleuropein) yielded the lowest values of the oleuropein treatments (*p* < 0.05). All oleuropein concentrations decreased WOB (*p* < 0.05), when compared with the control. The treatments with oleuropein showed no significant effects on the ratio of LIN and STR.

#### 3.2.2. Effects of Adding Oleuropein to Najdi Ram Semen Extender on Sperm Morphology, Acrosome Integrity, and Plasma Membrane Functionality in Cooled Sperms

As presented in Table 6, the normal sperm morphology, percentage of minor defects, acrosome integrity and HOST-positive sperms in cooled sperms were not affected by the addition of oleuropein at various levels. The percentage of major defects was significantly lower in all oleuropein groups (*p* < 0.05).

#### 3.2.3. Effects of Adding Oleuropein to Najdi Ram Semen Extender on Motility Kinetic and Characteristic Post-Thawing

The impacts of supplementation of the extender with oleuropein at varying concentrations on post-cryopreservation sperm motility kinetics, are presented in Table 7. Supplementation at levels of 1 and 2.5 mg/100 mL significantly improved VCL (*p* < 0.05), when compared to the control. The total motility was significantly increased at all studied concentrations of oleuropein, compared to the control group (*p* < 0.05). The 1 and 5 mg/100 mL concentrations of oleuropein increased the percentage of progressive motile spermatozoa (*p* < 0.001), compared with the control. Oleuropein supplementation at any studied concentration increased total motile spermatozoa, VSL, VAP, and LIN (*p* < 0.05). On the other hand, the treatments with oleuropein showed no significant effects on the ratio of WOB and STR.

#### 3.2.4. Effects of Adding Oleuropein to Najdi Ram Semen Extender on Sperm Vitality, Morphology, and Acrosome Integrity and Plasma Membrane Functionality Post-Thawing

Based on the obtained results regarding sperm morphology post-thawing, as presented in Table 8, all levels of oleuropein supplementation increased the percentage of minor defects (*p* < 0.01). Meanwhile, only 5 mg/100 mL oleuropein supplementation significantly improved the percentage of major defects compared with other groups. However, oleuropein supplementation at 2.5 and 5 mg/100 mL decreased acrosome integrity and normal sperm morphology (*p* < 0.001). Oleuropein supplementation at 1 or 2.5 mg/100 mL significantly increased sperm vitality (*p* < 0.001), and HOST was higher in all groups treated with oleuropein when compared to the control group (*p* < 0.001).

## 4. Discussion

It has been reported that egg yolk contains elevated concentrations of lipids, which in turn undergo lipolysis to form free fatty acids due to the process of ice crystallization during cryopreservation. The elevated levels of free fatty acids are responsible for the fluidity, flexibility, and receptor functionality at the spermatozoon plasma membrane [34]. Additionally, the lipid constituents of the spermatozoon cell membrane are partially responsible for plasma membrane microdomains that affect sperm motility. Consequently, diluents with elevated lipids could hamper the quality and membrane integrity of spermatozoa. Furthermore, the viscosity of the diluents used in semen extension may have effects on the motility of spermatozoa [35].

To the best of our knowledge, this is the first time that TRIS/egg yolk extenders supplemented with punicalagin or oleuropein have been used for the cryopreservation of Najdi ram semen. Diluters are crucial for the success of semen preservation, as they support the motility and viability of sperm for an extended time under ultra-low freezing temperatures. Therefore, many studies have focused on semen dilution and diluters for ram semen freezing. As stated above, egg yolk has suitable properties to protect spermatozoa against freezing–thawing damage, as it can set the spermatozoon plasma membrane. Cryoprotectants are substrates added supplementarily to semen diluters, which provide thermal protection for sperm cells during cryopreservation [36,37].

The percentage of motile spermatozoa is of capital importance in determining semen quality. The use of the CASA system minimizes the subjectivity of sperm motility assessment, and has had broad scientific and practical acceptance [28]. Sperm motility characteristics and kinematic characteristics have been widely used to evaluate semen quality and prediction of fertility outcome [38,39]. Furthermore, the CASA system gives a precise assessment of significant kinetic parameters [40].

The presented results revealed that the addition of punicalagin or oleuropein might preserve the motility—especially progressive motility—of spermatozoa. During cryopreservation, sperm cells are faced with irreparable injuries that hamper fertility. The benefit effects of punicalagin and oleuropein might be related to plasma membrane functionality, which allow the transport of vital molecules to the cytoplasm. The beneficial effects of punicalagin and oleuropein might be related to the plasma membrane, which allows for the transport of vital molecules to the cytoplasm. Furthermore, maintenance of the sperm plasma membrane is of utmost importance to achieve fusion between the spermatozoon and the ovum [41]. Accordingly, the HOST results suggested that supplementation of the used extender with punicalagin or oleuropein could enable some resistance of the spermatozoa plasma membrane during cryopreservation.

Sperm cells typically present an elevated proportion of PUFAs and a lower ratio of cholesterol to phospholipids, which make sperm cells more sensitive to ROS resulted from lipid peroxidation [42,43]. Spermatozoa are tolerant to ROS and can neutralize them via the natural antioxidants (such as glutathione reductase, glutathione peroxidase, catalase, and superoxide dismutase) present in seminal plasma, membranes, and cytoplasm [44,45,46]. However, the capability of spermatozoa to synthesize these antioxidants may be inadequate, especially in the presence of oxidative stressors beyond their capacity [44]; moreover, the use of a dilution process during semen cryopreservation dilutes the natural existing antioxidants in the seminal plasma, consequently diminishing their antioxidative capability. Accordingly, supplementation with exogenous antioxidants is essential to maintain and enhance the function of spermatozoa during cryopreservation. Unfortunately, the cryopreservation process deleteriously affects the concentrations of these endogenous antioxidants [47,48]. Accordingly, semen diluters should be supplemented with certain antioxidants, in order to minimize the harmful effects of oxidative stress during cryopreservation. Thus, in this study, punicalagin or oleuropein—as antioxidant agents—were used as supplements in the semen diluter used for ram semen freezing.

Punicalagin—a significant constituent of pomegranate polyphenols—has been demonstrated in numerous studies to possess antioxidant qualities. Punicalagin has been shown to prevent diabetic liver damage through increasing the activity of antioxidant enzymes and mitophagy [49]. Moreover, pomegranate peel extract, which is rich in punicalagin, has been found to possess substantial antioxidant activity [50]. Punicalagin has also been found to have antimicrobial and antioxidant potential [51]. Mansour et al. [52] reported that the administration of pomegranate extract containing punicalagin enhanced sperm quality parameters and antioxidant activity in adult male Wistar rats; the same benefits were found in human men [53]. Similar results have been reported in rooster semen; moreover, resistance against lipid peroxidation during liquid storage was found to be increased [54]. In addition, punicalagin has been shown to exert anti-inflammatory and microbiota-modulating effects in murine colitis models [55], as well as attenuating ventricular remodeling after acute myocardial infarction through regulation of the NLRP3/caspase-1 pathway [56].

Oxidative stress has been reported to have negative impacts on sperm cryo-resistance, due to plasma and acrosome membrane impairment and nuclear damage [13]. The results of other studies conducted in rams [57], goats [58], and horses [59] have revealed strong correlations between ROS and viability, sperm motility, and plasma membrane integrity [60]; in particular, these effects can be attributed to peroxidation of the lipid content of the sperm membrane. Oleuropein has antioxidant and antimicrobial properties. In this study, adding oleuropein to TRIS diluent improved ram semen quality. Alirezaei et al. [61] elucidated that oleuropein possesses beneficial antioxidant effects in the context of ethanol-induced sperm toxicity and, accordingly, improved sperm motility and plasma membrane integrity. In a study in rats, olive leaf extract (which includes oleuropein) was found to increase the quality and total antioxidant capacity of sperm while decreasing malondialdehyde levels in the testis [62]. Olea europaea leaf extract is regarded to have high levels of polyphenols, may exert protective effects against oxidative stress induced by H_2_O_2_ in human sperm, and has been reported to increase the quality of sperm post-thawing [21]. Oleuropein has potential protective mechanisms against various health problems, including inflammation, microbial infections, and oxidative stress [63]. Oleuropein has potential antioxidant activities and may be used to improve velocity and total sperm motility parameters in ram semen post-thaw [64].

The patterns of sperm motility (i.e., rectilinear velocity, curvilinear velocity, average path velocity, linearity coefficient, and straightness index) are the best indices for the determination of the capacity and hyperactivity of sperm, reflecting their capability to enter the oocytes’ zona pellucida [65]. However, the sperm velocity is related to the total percentage of progressively motile spermatozoa; hence, progressive motility is considered a critical quality parameter [66]. The addition of the antioxidants considered in this study resulted in an effective improvement in sperm velocity. Our findings correspond well with previous research [24,25,54,64]. Zhang et al. [67] have clarified that diluent supplemented with punicalagin boosted the quality of ram sperm preserved at 4 °C by increasing the antioxidant capacity and decreasing oxidative stress. Finally, this study demonstrated the potential for the use of exogenous antioxidants in semen diluents, providing a reference for improving ram semen quality.

## 5. Conclusions

This study aimed to investigate the effects of adding different levels of punicalagin or oleuropein to TRIS/egg yolk diluent on the quality of frozen–thawed semen from Najdi rams. It was found that supplementation of the extender with punicalagin or oleuropein improved the viability, total motility and progressive motility, and the patterns of motility in spermatozoa. In particular, punicalagin supplementation improved acrosome and plasma membrane integrity. Finally, adding punicalagin (0.1 mg/100 mL) or oleuropein (1 mg/100 mL) to TRIS diluent improved the quality of frozen–thawed semen from rams.

## Figures and Tables

**Table 1:** Effects of adding punicalagin to Najdi ram semen extender on motility kinetics and characteristics of sperm in cooled sperms. (Mean ± standard error).

Items/ in Cooled Sperms	Control	Punicalagin (mg/100 mL)			*p*-Value
	0.0 mg/100 mL Punicalagin	P1: 0.1	P2: 0.5	P3: 1	
TMS (%)	88.78 ± 2.57 ^b^	97.00 ± 0.05 ^a^	87.30 ± 3.11 ^b^	86.30 ± 0.57 ^b^	<0.05
PMS (%)	49.72 ± 1.52 ^b^	67.05 ± 1.24 ^a^	41.30 ± 0.05 ^b^	40.05 ± 0.77 ^b^	<0.001
VCL (µm/s)	78.90 ± 0.60 ^b^	82.23 ± 0.25 ^a^	75.06 ± 0.10 ^b^	74.22 ± 0.14 ^b^	<0.001
VSL (µm/s)	30.14 ± 1.27 ^b^	40.50 ± 0.15 ^a^	40.30 ± 0.13 ^a^	38.12 ± 0.15 ^a^	<0.001
VAP (µm/s)	47.27 ± 1.30 ^b^	57.11 ± 0.35 ^a^	51.58 ± 0.05 ^a^	49.33 ± 0.15 ^a^	<0.001
LIN (%)	38.98 ± 1.25 ^b^	49.13 ± 0.07 ^a^	53.88 ± 0.24 ^a^	52.02 ± 0.11 ^a^	<0.001
WOB (%)	60.43 ± 1.11 ^b^	69.11 ± 0.17 ^a^	68.97 ± 0.16 ^a^	66.98 ± 0.09 ^a^	<0.001
STR (%)	64.25 ± 0.89 ^b^	71.10 ± 0.17 ^a^	78.12 ± 0.17 ^a^	77.57 ± 0.06 ^a^	<0.001

^a,b^ Means in the same row with different superscripts are significantly different (*p* < 0.05). TMS: Total motile spermatozoa; PMS: Progressive motile spermatozoa; VCL: Curvilinear velocity (μm/s); VSL: rectilinear velocity (μm/s); VAP: average path velocity (μm/s); LIN: linearity coefficient; STR: straightness index; WOB: Wobble of the curvilinear trajectory.

**Table 2:** Effects of adding punicalagin to Najdi ram semen extender on sperm morphology, acrosome integrity, and plasma membrane integrity in cooled sperms. (Mean ± standard error).

Items/ in Cooled Sperms	Control	Punicalagin (mg/100 mL)			*p*-Value
	0.0 mg/100 mL Punicalagin	P1: 0.1	P2: 0.5	P3: 1	
Normal (%)	81.26 ± 4.45	75.21 ± 0.28	83.16 ± 2.81	81.09 ± 0.69	0.391
Major defect (%)	8.37 ± 1.84	6.77 ± 0.29	5.64 ± 1.02	5.79 ± 0.33	0.419
Minor defect (%)	10.28 ± 2.66	18.01 ± 0.58	11.18 ± 1.80	13.11 ± 0.35	0.077
Acrosome integrity (%)	87.50 ± 3.68	91.27 ± 0.34	93.07 ± 1.34	92.77 ± 0.21	0.357
HOST (%)	78.23 ± 1.03 ^b^	67.04 ± 2.02 ^b^	66.53 ± 1.48 ^b^	82.72 ± 1.12 ^a^	<0.001

^a,b^ Means in the same row with different superscripts are significantly different (*p* < 0.05). HOST: hypo-osmotic swelling test.

**Table 3:** Effects of adding punicalagin to Najdi ram semen extender on motility kinetics and characteristics post-thawing. (Mean ± standard error).

Items/ Post-Thawing	Control	Punicalagin (mg/100 mL)			*p*-Value
	0.0 mg/100 mL Punicalagin	P1: 0.1	P2: 0.5	P3: 1	
TMS (%)	27.65 ± 2.65 ^b^	64.25 ± 0.14 ^a^	48.00 ± 0.05 ^a^	39.30 ± 0.92 ^a^	<0.001
PMS (%)	13.43 ± 0.03 ^b^	37.30 ± 0.28 ^a^	13.35 ± 0.08 ^b^	8.10 ± 0.23 ^b^	<0.001
VCL (µm/s)	78.79 ± 0.24	81.27 ± 0.04	69.06 ± 6.02	76.09 ± 0.14	0.091
VSL (µm/s)	44.18 ± 0.10 ^b^	53.15 ± 0.12 ^a^	46.75 ± 2.15 ^b^	39.77 ± 0.68 ^b^	<0.001
VAP (µm/s)	55.34 ± 0.07 ^b^	62.81 ± 0.06 ^a^	52.33 ± 2.05 ^b^	51.17 ± 0.48 ^b^	<0.001
LIN (%)	54.82 ± 0.13 ^b^	64.60 ± 0.23 ^a^	58.36 ± 3.27 ^b^	52.39 ± 0.69 ^b^	<0.005
WOB (%)	69.73 ± 0.09 ^a^	76.65 ± 0.14 ^a^	51.41 ± 1.42 ^b^	67.69 ± 0.48 ^a^	<0.05
STR (%)	78.46 ± 0.11 ^b^	84.22 ± 0.14 ^a^	73.93 ± 0.98 ^b^	77.41 ± 0.48 ^b^	<0.001

^a,b^ Means in the same row with different superscripts are significantly different (*p* < 0.05). TMS: Total motile spermatozoa; PMS: Progressively motile spermatozoa; VCL: Curvilinear velocity (μm/s); VSL: rectilinear velocity (μm/s); VAP: average path velocity (μm/s); LIN: linearity coefficient; STR: straightness index; WOB: Wobble of the curvilinear trajectory.

**Table 4:** Effects of adding punicalagin to Najdi ram semen extender on sperm vitality, morphology, and acrosome and plasma membrane integrity post-thawing. (Mean ± standard error).

Items/ Post-Thawing	Control	Punicalagin (mg/100 mL)			*p*-Value
	0.0 mg/100 mL Punicalagin	P1: 0.1	P2: 0.5	P3: 1	
Sperm vitality (%)	30.45 ± 1.65 ^b^	42.42 ± 3.55 ^a^	13.47 ± 2.46 ^b^	19.23 ± 1.10 ^b^	<0.001
Normal (%)	90.37 ± 0.47	84.54 ± 0.23	85.34 ± 0.90	84.55 ± 2.47	0.115
Major defect (%)	4.33 ± 0.39	2.94 ± 0.48	2.88 ± 0.19	3.31 ± 0.61	0.257
Minor defect (%)	5.46 ± 0.09 ^b^	12.51 ± 0.25 ^a^	11.77 ± 0.71 ^a^	12.12 ± 1.98 ^a^	<0.05
Acrosome integrity (%)	94.70 ± 0.95	96.46 ± 0.59	96.56 ± 0.27	95.96 ± 0.83	0.331
HOST (%)	60.69 ± 2.40 ^b^	65.50 ± 2.24 ^a^	64.25 ± 1.92 ^a^	68.44 ± 1.80 ^a^	<0.001

^a,b^ Means in the same row with different superscripts are significantly different (*p* < 0.05). HOST: hypo-osmotic swelling test.

**Table 5:** Effects of adding oleuropein to Najdi ram semen extender on motility kinetics and characteristics in cooled sperms. (Mean ± standard error).

Items/ in Cooled Sperms	Control	Oleuropein (mg/100 mL)			*p*-Value
	0.0 mg/100 mL Oleuropein	O1: 1	O2: 2.5	O3: 5	
TMS (%)	91.45 ± 2.97	94.00 ± 1.90	81.05 ± 5.74	86.40 ± 0.63	0.104
PMS (%)	51.25 ± 1.76	69.70 ± 1.67	50.25 ± 1.65	46.85 ± 1.81	0.070
VCL (µm/s)	79.50 ± 0.69 ^b^	87.63 ± 2.56 ^a^	83.14 ± 2.04 ^ab^	80.97 ± 0.09 ^b^	<0.05
VSL (µm/s)	31.41 ± 1.46	27.92 ± 1.34	29.74 ± 1.46	28.20 ± 0.85	0.284
VAP (µm/s)	48.57 ± 1.50 ^a^	48.13 ± 0.07 ^a^	46.60 ± 0.49 ^ab^	44.52 ± 0.21 ^b^	<0.05
LIN (%)	40.23 ± 1.44	32.74 ± 2.30	36.79 ± 2.71	35.90 ± 1.04	0.145
WOB (%)	61.55 ± 1.28 ^a^	55.62 ± 1.53 ^b^	56.85 ± 0.89 ^b^	55.74 ± 0.29 ^b^	<0.05
STR (%)	65.14 ± 1.02	58.58 ± 2.55	64.42 ± 3.74	64.12 ± 1.54	0.283

^a,b^ Means in the same row with different superscripts are significantly different (*p* < 0.05). TMS: Total motile spermatozoa; PMS: Progressive motile spermatozoa; VCL: Curvilinear velocity (μm/s); VSL: rectilinear velocity (μm/s); VAP: average path velocity (μm/s); LIN: linearity coefficient; STR: straightness index; WOB: Wobble of the curvilinear trajectory.

**Table 6:** Effects of adding oleuropein to Najdi ram semen extender on sperm morphology, acrosome integrity, and plasma membrane integrity in cooled sperms. (Mean ± standard error).

Items/ in Cooled Sperms	Control	Oleuropein (mg/100 mL)			*p*-Value
	0.0 mg/100 mL Oleuropein	O1: 1	O2: 2.5	O3: 5	
Normal (%)	82.31 ± 3.60	86.19 ± 0.68	91.61 ± 0.11	89.04 ± 0.23	0.134
Major defect (%)	7.74 ± 1.56 ^a^	5.29 ± 0.26 ^b^	3.39 ± 0.07 ^b^	1.81 ± 0.24 ^b^	<0.05
Minor defect (%)	9.94 ± 2.09	8.51 ± 0.41	4.99 ± 0.19	9.14 ± 0.48	0.213
Acrosome integrity (%)	89.00 ± 3.22	93.83 ± 0.36	96.29 ± 0.07	97.85 ± 0.52	0.089
HOST (%)	72.01 ± 1.02	76.94 ± 1.09	74.79 ± 4.21	69.50 ± 2.67	0.356

^a,b^ Means in the same row with different superscripts are significantly different (*p* < 0.05). HOST: hypo-osmotic swelling test.

**Table 7:** Effects of adding oleuropein to Najdi ram semen extender on motility kinetics and characteristics post-thawing. (Mean ± standard error).

Items/ Post-Thawing	Control	Oleuropein (mg/100 mL)			*p*-Value
	0.0 mg/100 mL Oleuropein	O1: 1	O2: 2.5	O3: 5	
TMS (%)	25.07 ± 2.10 ^b^	42.75 ± 3.22 ^a^	49.20 ± 3.00 ^a^	70.24 ± 5.19 ^a^	<0.001
PMS (%)	13.46 ± 0.32 ^b^	30.26 ± 4.72 ^a^	23.25 ± 0.89 ^ab^	47.13 ± 4.57 ^a^	<0.001
VCL (µm/s)	78.79 ± 0.08 ^b^	81.00 ± 0.80 ^a^	81.33 ± 0.03 ^a^	79.61 ± 0.77 ^ab^	<0.05
VSL (µm/s)	44.28 ± 0.14 ^b^	50.98 ± 2.44 ^a^	51.11 ± 0.11 ^a^	49.70 ± 1.85 ^a^	<0.05
VAP (µm/s)	55.41 ± 0.10 ^b^	61.18 ± 1.70 ^a^	61.44 ± 0.39 ^a^	58.72 ± 1.96 ^a^	<0.05
LIN (%)	54.96 ± 0.12 ^b^	61.50 ± 2.54 ^a^	61.25 ± 0.49 ^a^	62.39 ± 2.19 ^a^	<0.05
WOB (%)	69.83 ± 0.07	74.62 ± 1.42	74.58 ± 0.28	73.70 ± 1.89	0.640
STR (%)	78.54 ± 0.08	82.11 ± 1.92	81.87 ± 1.08	84.59 ± 1.53	0.580

^a,b^ Means in the same row with different superscripts are significantly different (*p* < 0.05). TMS: Total motile spermatozoa; PMS: Progressive motile spermatozoa; VCL: Curvilinear velocity (μm/s); VSL: rectilinear velocity (μm/s); VAP: average path velocity (μm/s); LIN: linearity coefficient; STR: straightness index; WOB: Wobble of the curvilinear trajectory.

**Table 8:** Effects of adding oleuropein to Najdi ram semen extender on sperm vitality, morphology, and acrosome and plasma membrane integrity post-thawing. (Mean ± standard error).

Items/ Post-Thawing	Control	Oleuropein (mg/100 mL)			*p*-Value
	0.0 mg/100 mL Oleuropein	O1: 1	O2: 25	O3: 5	
Sperm vitality (%)	28.65 ± 2.14 ^b^	47.20 ± 2.53 ^a^	45.47 ± 1.24 ^a^	34.68 ± 1.27 ^b^	<0.001
Normal (%)	89.88 ± 0.56 ^a^	88.13 ± 0.29 ^a^	85.29 ± 0.31 ^b^	84.39 ± 0.15 ^b^	<0.001
Major defect (%)	4.74 ± 0.46 ^b^	5.13 ± 0.12 ^b^	5.81 ± 0.31 ^b^	8.51 ± 0.33 ^a^	<0.001
Minor defect (%)	5.37 ± 0.10 ^b^	6.73 ± 0.16 ^a^	8.89 ± 0.63 ^a^	7.09 ± 0.48 ^a^	<0.002
Acrosome integrity (%)	94.71 ± 0.54 ^a^	94.16 ± 0.16 ^a^	93.15 ± 0.33 ^b^	89.92 ± 0.36 ^b^	<0.001
HOST (%)	55.87 ± 2.48 ^b^	69.85 ± 1.11 ^a^	68.66 ± 1.36 ^a^	65.07 ± 1.32 ^a^	<0.001

^a,b^ Means in the same row with different superscripts are significantly different (*p* < 0.05). HOST: hypo-osmotic swelling test.

## Data Availability

The data that support the findings of this study are available on request from the corresponding author.
